# Calculi of the Prostate Gland

**Published:** 1846-08

**Authors:** 


*Calculi of the Prostate Gland.*—A discussion which occurred recently at the "Societé de Chirurgie," on prostatic calculi, and which is reported by the *Gazette des Hôpitaux*, elicited the following remarks on the subject:—

M. Lenoir stated that a patient, fifty-five years of age, had been addressed to him by a provincial surgeon, under the impression that he was labouring under vesical calculus. On introducing the sound, he found an obstacle which gave a clear sound, and which he thought was a vesical calculus, but on examining digitally by the rectum, he failed to recognise its presence. On exercising pressure, however, on the prostate, he caused the escape of about fifteen small calculi. They were of a dark yellow colour, and presented facet surfaces; burnt, they gave a decided animal odour. The patient, who, when he entered the hospital, had all the symptoms of serious vesical catarrh, left nearly well. A few months later, he was again sent to Paris, under the idea that he was labouring from vesical calculus, and a number of small stones were again emitted, by pressure of the prostate. Vesical catarrh was present, as on the first occasion. M. Lenoir thought that the calculi were formed in the ejaculatory ducts, and that it was because they occupied the orifice, that these produced, when touched with the sound, the sensation of a stone in the bladder.

M. Nelaton had met with a case at the Hotel Dieu, similar to the one of M. Lenoir. The friction of the sound over a hard substance in the region of the prostate had led him to recognise the presence of prostatic calculi. He managed to withdraw several by means of lithotritic instruments, and the patient left apparently cured. Two months afterwards he returned with the same symptoms, indicating prostatic calculi, and, in addition, with a vesical calculus. He was not able to lay hold of the latter, in order to crush it, and was obliged to perform the operation of lithotomy. On scratching the surface of the incised prostate with his nail, he managed to make several calculi fall, similar to those described by M. Lenoir. The patient was cured. M. Michon, M. Guersant, and M. Laugier, thought that prostatic calculi were not rare; M. Malgaigne was of a contrary opinion.—*London Lancet.*

